# Investigation of novel combination therapy for age-related macular degeneration on ARPE-19 cells

**DOI:** 10.3389/fddev.2024.1337686

**Published:** 2024-04-19

**Authors:** Madhuri Dandamudi, Peter McLoughlin, Gautam Behl, Lee Coffey, Anuj Chauhan, David Kent, Sweta Rani, Laurence Fitzhenry

**Affiliations:** ^1^ Ocular Therapeutics Research Group, Pharmaceutical and Molecular Biotechnology Research Centre, Department of Science, South East Technological University, Waterford, Ireland; ^2^ Eirgen Pharma Ltd, Waterford, Ireland; ^3^ Department of Chemical and Biological Engineering, Colorado School of Mines, Colorado, CO, United States; ^4^ The Vision Clinic, Kilkenny, Ireland

**Keywords:** AMD, macular degeneration, combination therapy, ARPE-19, triamcinolone acetonide, quercetin, corticosteroid, flavonoid

## Abstract

Age-related macular degeneration (AMD) is a multifactorial degenerative disease characterised by the gradual loss of central vision in individuals aged more than 50 years. There is currently no cure for this disease, but treatment can delay its progression. Consequently, there is an urgent need for the development of both new and cost-effective therapeutics. In this study, a novel combination of a corticosteroid and flavonoid was investigated on human retinal pigment epithelial cell lines to explore its potential pharmacological effect on AMD. Combination therapies, such as anti-VEGF (vascular endothelial growth factor) agents combined with photodynamic therapy and anti-VEGF agents in conjunction with corticosteroids, have been utilized previously and are known to be effective. However anti-VEGF injections are associated with serious side effects and are costly. Various disease conditions associated with AMD were stimulated on human retinal cells, which were then exposed to different concentrations of triamcinolone acetonide (TA) and quercetin (QCN) individually and in combination. This investigation aimed to assess their potential for the treatment of AMD. The combination of TA and QCN demonstrated a superior anti-inflammatory effect, as TA and QCN primarily act on different inflammatory signaling pathways. Furthermore, in terms of anti-VEGF activity, both drugs exert their effects through different mechanisms: QCN inhibits kinase pathways leading to the deactivation of VEGF receptors, whereas TA destabilises VEGF mRNA, resulting in increased suppression of VEGF-C with combination treatments. The anti-oxidant assay yielded similar outcomes, demonstrating a synergetic effect when treated with combination drugs. These findings collectively suggest TA and QCN as a promising combination therapy for targeting AMD with multiple pathological conditions.

## 1 Introduction

**FIGURE 1:**
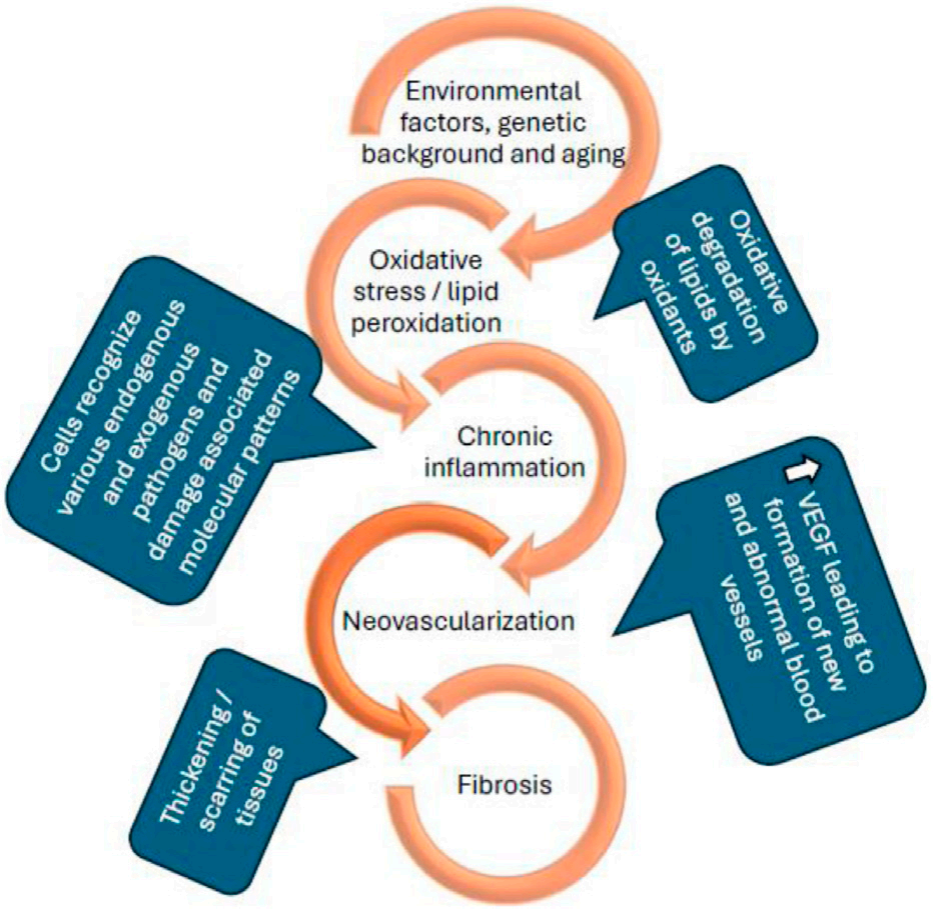
Schematic representation of the pathological conditions of age-related macular degeneration (P. Zhou and Xiao-Li, 2014; Wong et al., 2022; Kaarniranta et al., 2013).

Age-related macular degeneration (AMD) is an accelerating blinding disease with no cure. Current available treatments can only slow down disease progression. Initial investigations led to wet AMD being considered as a vascular disease, primarily involving angiogenesis and choroidal neovascularization (CNV) (Brown et al., 2011). However, it is now considered as a complicated multifactorial disorder with various non-vascular components (Rozing et al., 2020). Current anti-VEGF (vascular endothelial growth factor) agents control CNV which occurs in the later stages of AMD. At this stage, branching and development of new blood vessels evolve from the choroid, which later reach the RPE and damage central vision. While current treatments are effective, they are associated with serious side effects, such as retinal detachment (Saito et al., 2013), retinal hemorrhage (Day et al., 2011), endophthalmitis (Fileta et al., 2014), an increase in intraocular pressure (Sternfeld et al., 2020) and, as stated above, do not cure the condition. With increasing evidence supporting the complexity of AMD pathology, the investigation of new therapeutic targets and combination therapies that can target multiple pathologies of the disease (as seen in Figure 1) has received increased attention.

A synergistic effect is observed when two or more drugs interact and produce an enhanced effect when compared to the sum of their individual effects (Foucquier and Guedj, 2015). A synergetic interaction allows for a lower dose of individual drugs, which may reduce adverse effects. Combination therapies such as anti-VEGF agents together with photodynamic therapy (Hatz et al., 2017), and anti-VEGF agents along with corticosteroids (Kuppermann et al., 2015) have been used previously and are known to be effective. These anti-VEGF injections have serious side effects, and monoclonal antibodies (current anti-VEGF agents) are expensive. There is an unmet need to develop non-invasive and effective treatment options for AMD, which can be self-administered by patients. In the current study, a novel combination of a corticosteroid (triamcinolone acetonide) and flavonoid (Quercetin) was investigated on human retinal pigment epithelial cell lines to determine the potential pharmacological effect on AMD. Triamcinolone acetonide (TA) is one of the foremost compounds evaluated for the treatment for multiple back of the eye diseases such as, macular edema, uveitis, AMD, and vitreoretinopathy. The initial use of intravitreal TA to treat exudative AMD resulted in significant reduction of CNV but in long-term use, TA as a monotherapy had no effect on visual acuity loss (despite significant anti-angiogenic effects beyond 3 months of treatment). Hence a combination of TA and photodynamic therapy was performed, which acted synergistically and reduced the number of re-treatments (Sarao et al., 2014). In the present study, TA was investigated in combination with quercetin (QCN), considering its reported anti-oxidant and anti-VEGF properties (F. Li et al., 2015; Shao et al., 2019). Some of the crucial pathological conditions of AMD were stimulated on human retinal cells (oxidative stress, inflammation, and VEGF secretion) and exposed to different concentrations of TA and QCN both individually and in combination, in an attempt to investigate their potential for the treatment of AMD. Initially, non-toxic concentrations of the individual drugs and combinations were identified using the *in-vitro* cytotoxicity assay on ARPE-19 (Human adult retinal pigment epithelium-19. Following the cytotoxicity study ELISA (enzyme-linked immunosorbent assay) was performed to investigate the effect of treatments on the secretion of chosen inflammatory and angiogenic cytokines. The effect of drugs on oxidative was observed in *in-vitro* conditions through a chemical assay and flow cytometry.

## 2 Materials and methods

### 2.1 Materials

Adult retinal pigment epithelium-19 (CRL-2302™) and Fetal Bovine Serum (ATCC-30-2025, EU Approved, South American Origin) were purchased from LGC standards Ltd, UK. DMEM/F-12 cell culture media (Gibco™ 31330038), Trypsin (Gibco™ 25300054), Penicillin-Streptomycin (Gibco™ 15070063) and p-nitrophenyl phosphate (PNPP) were procured from Fisher Scientific, Ireland. Dichlorofluorescin diacetate (DCFH-DA) and 2,2-diphenyl-1-picryl-hydrazyl-hydrate (DPPH) were purchased from Sigma Aldrich, Ireland. IL-6, IL-8, MCP-1, and VEGF-C ELISA kits were purchased from Assay genie, Ireland. Triamcinolone acetonide (TA) (MW: 434.50 g/mol with purity ˃ 99%) and Quercetin (QCN) (MW: 302.24 g/mol with purity >99%) were procured from Carbosynth Ltd, UK. 96, 24 and six well plates and serological pipettes were procured from Greiner bio-one, Cruinn diagnostics, Ireland.

The following equipment was used throughout the experimental work: Refrigerated centrifuge (Sigma 3-18KS, Focus scientific, Ireland), plate reader (HTS Plate Reader-MSD Model 1,250 Sector Imager, US), inCu safe cell culture incubator (Davidson and Hardy Ltd, Ireland), - 80 °C chest freezer (Medical supply company, Ltd, Ireland), flow cytometer (Cytomics FC500, Beckman Coulter, United States), Memmert–Water Bath (Mason technology, Ireland), Airstream^®^ Class II Biological Safety Cabinet (ESCO, Mason technology, Ireland), and Classic Vortex Mixer (Fisherbrand™, Ireland).

### 2.2 Cell culture

ARPE-19 cell line was cultured using cell culture media containing a 1:1 mixture of DMEM and F-12 nutrient mixture. The media was supplemented with 10% FBS and 1% penicillin-streptomycin antibiotic mixture. During the culture, cells were maintained at 37 °C with 5% CO_2_ in a humidified incubator and sub-cultured with trypsin upon confluency.

### 2.3 Cytotoxicity evaluation of individual drugs and drug combinations

Cytotoxicity evaluation of the drugs, both individually and in combination, was performed using the acid phosphatase assay (APA) (Bojarska et al., 2022). Initial stock solutions of the drugs were prepared in DMSO, and the working solutions were prepared and diluted using cell culture media. The cytotoxicity assay was performed on TA and QCN from 5 to 250 μM and 1–250 μM, respectively (Supplementary Figure S1). After investigating individual drugs, drug combinations were tested. Each concentration of TA at 10, 25, 50, 75 and 100 µM was tested in combination with all the concentrations of QCN (5, 10, 15 and 20 µM). Initially ARPE-19 cells were seeded onto a 96-well plate at a seeding density of 5,000 cells/well containing 100 µL of cell culture media and cultured for 24 h. After 24 h, treatments were added to the wells and incubated for the period of the study (24 h and 48 h). After the treatment period, media was removed, and cells were washed twice with PBS. Upon washing, 100 µL of 10 mM PNPP substrate dissolved in 0.1 M sodium acetate buffer was added to the wells and incubated for 2 h. Finally, 50 µL of stop solution (1 M sodium hydroxide) was added and the plate was analysed in the plate reader at 405 nm.

### 2.4 Anti-inflammatory and anti-VEGF activities on ARPE-19 using ELISA

The human IL-6, IL-8, MCP-1, and VEGF-C ELISA kits were used according to the manufacturer’s protocol to investigate the cytokine secretions in the cell supernatants. The individual drugs and combination drugs were tested at similar concentrations as those used for the cytotoxicity study in Section 2.3. Cells were seeded onto a 24-well plate at a seeding density of 3 × 10^4^ cells/well with 500 µL of cell culture media and cultured for 24 h. Cells were kept overnight in low serum media (with 1% FBS) to synchronise their growth phase before stimulating inflammation. After 24 h cells were stimulated with 10 μg/mL LPS to induce inflammation for a duration of 24 h. Upon stimulation, cells were exposed to various treatments of drugs alone and in combination for 24 h. After the treatment duration media conditioned by treated cells were collected and analysed for the cytokines and VEGF-C secretions using the ELISA kits. ELISA was performed according to the manufacturer’s protocol.

### 2.5 Antioxidant activity

#### 2.5.1 2,2-Diphenyl-1-picrylhydrazyl (DPPH) assay

The DPPH assay was performed in a 96-well plate where 20 µL of methanolic DPPH solution was added to 180 µL of methanolic solutions of treatments (Skouta et al., 2018). Based on the outcome from anti-inflammatory studies, the higher concentrations of TA and QCN, both individually and in combination were investigated for antioxidant activity (TA 75, 100 µM and QCN 15, 20 µM). This reaction mixture was incubated for 30 min in darkness at room temperature and the absorbance was measured at 517 nm using a microplate reader.

#### 2.5.2 Dichloro-dihydro-fluorescein diacetate (DCFH-DA) assay

ARPE-19 cells were seeded onto 6-well plates at a seeding density of 25 × 10^4^ cells/well and incubated for 24 h. At the 24 h time point cells were stimulated to induce oxidative stress. The stimulants, LPS and H_2_O_2_ were tested at concentrations 10, 20, 40 μg/mL and 100, 200, 300 μM, respectively. Following stimulation, cells were exposed to similar concentrations of TA and QCN as used for the DPPH assay in Section 2.5.1 (both individually and in combination) for 24 h and after the treatment period cells were incubated with 20 µM DCFH-DA dye (Behl et al., 2013). Upon incubation with dye, cells were detached using trypsin and resuspended in PBS for analysis using flow cytometry.

### 2.6 Statistics

Statistical differences between the treatments were assessed using Student’s t-test. The results were considered statistically significant if the *p*-value was less than 0.05. All statistical analysis were performed using both excel and GraphPad^®^ software.

## 3 Results and discussion

### 3.1 Drugs cytotoxicity study

Cytotoxicity studies are the important first step to assess the safety of drug molecules during the developmental stage before testing them on animals. APA was utilised to assess the cytotoxicity effect of the treatments. The hydrolysis of the p-nitrophenyl phosphate substrate by the intracellular acid phosphatases in viable cells results in the formation of p-nitrophenol. As outlined in Section 2.3, the absorbance of p-nitrophenol was measured at 405 nm, which is directly proportional to cell viability. Before investigating the combination of drugs on ARPE-19 cells, individual drugs were studied. The concentrations were selected from previously published studies (with higher concentrations included to establish toxic and non-toxic concentrations) (Saviranta et al., 2011; Hytti et al., 2015; Hirani et al., 2016; Cheng et al., 2019; HSIAO et al., 2021). Both TA and QCN were evaluated between 10 and 250 µM concentrations for 24 and 48 h periods. The viability of cells was not affected with exposure to TA from 10 to 250 µM (cell viability >80%) and no changes in cell morphology were observed as seen in Supplementary Figure S1A. Whereas QCN exhibited a decrease in cell viability with increase in concentration. The QCN concentration up to 25 µM displayed more than 80% cell viability but the higher concentrations exhibited a toxic effect on cells (SF 1(b)). Given these results, a further study focused on lower concentrations of QCN, 1–100 µM.

**FIGURE 2:**
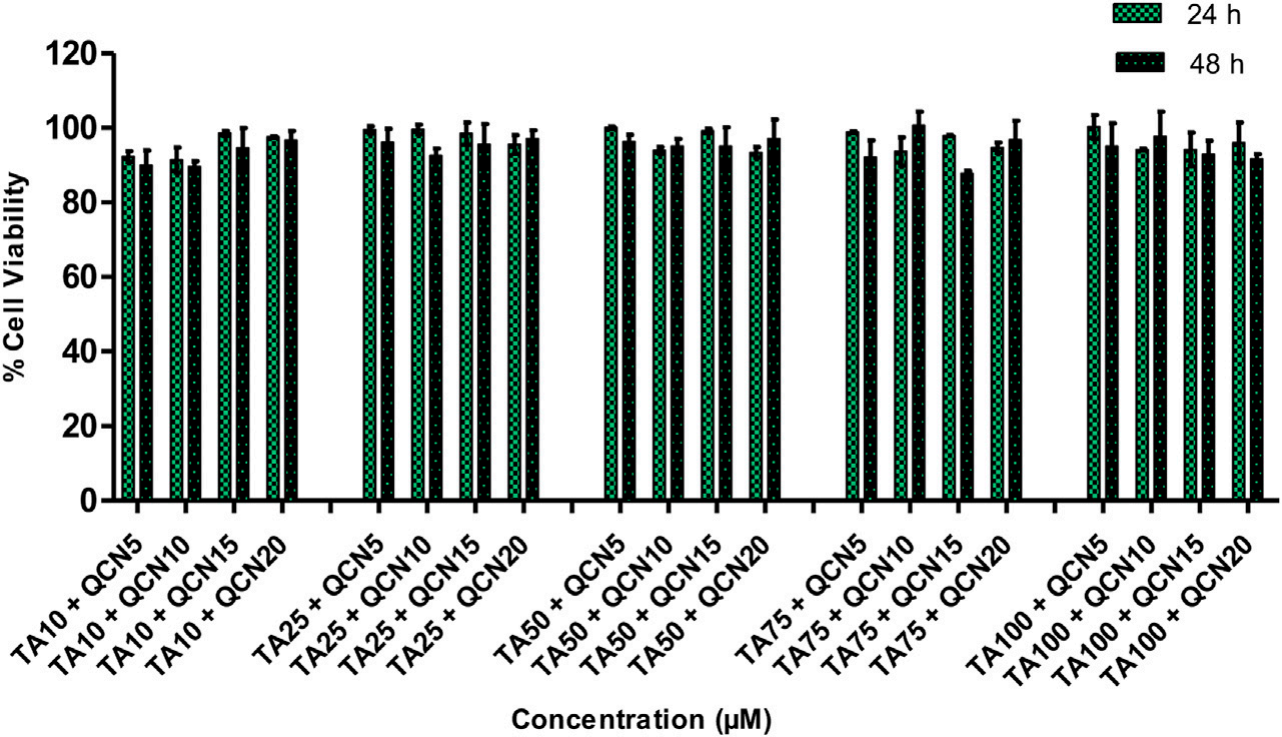
% Cell viability of a combination of drugs (TA and QCN: TA + QCN) on ARPE-19 cells. Data points represent the average ±SD of n = 3 biological replicates.

The viability of the ARPE-19 cells was not significantly changed (*p* > 0.05) by the treatment with QCN from 1 to 30 µM. Nonetheless, QCN concentrations from 40 to 100 µM significantly decreased the viability of cells with *p*-value <0.05 (SF 2). These results relate to a previous study by Wang et al., they evaluated the effect of QCN on high glucose-induced injury in ARPE-19, the viability of the cells was decreased with QCN concentration greater than 30 µM (Wang et al., 2020). Similar results were noticed by Cheng et al., who investigated the effect of QCN on inflammatory cytokines and chemokines in ARPE-19 cells (Cheng et al., 2019). According to the above-mentioned literature, QCN concentrations up to 20 µM proved to be non-toxic with an anti-inflammatory effect in *in-vitro* conditions. Previous investigations discovered that flavonoids such as QCN, luteolin, apigenin, etc., exhibited beneficial effects at low concentrations but are toxic at higher concentrations on human cell lines with the structure-cytotoxicity relationship still unclear (Atsuo et al., 2005). The QCN concentration from 1 to 20 µM demonstrated more than 90% cell viability and proved to be safe on human retinal cells. Based on these results and effective concentrations of the drugs on the pathology of AMD in previous studies (Cheng et al., 2019; HSIAO et al., 2021); TA 10, 25, 50, 75 and 100 µM and QCN from 5, 10, 15 and 20 µM were chosen to be studied in combination. Drug combinations showed no synergetic toxicity on ARPE-19 cells or changes to cell morphology (Figure 2).

All the chosen combination concentrations demonstrated more than 85% cell viability, similar to the individual drugs. The cytotoxicity assay estimates the loss of cellular and intercellular functions or structure, which includes cytotoxicity effects. Hence, this provided insight into any potential tissue/cell injury and irritation during application. This *in-vitro* cytotoxicity study suggested that the individual drugs and in combination were safe on human retinal cell lines.

### 3.2 Anti-inflammatory activity studies using ARPE-19 cells

Multiple studies on biological samples from AMD patients and studies which assessed physiological conditions during the disease state supports the conclusion that AMD is a multifactorial disorder, which involves RPE dysfunction and damage to photoreceptor cells (mainly due to inflammatory conditions and oxidative stress) (Fritsche et al., 2014; Singh et al., 2021; Deng et al., 2022). RPE plays a crucial role in the formation of the blood-retinal barrier (BRB), establishment of ocular immune privilege and in secretion of immunomodulatory factors to monitor immunogenic inflammation. In the case of AMD, damage to RPE effects ocular immune tolerance distorting BRB, downregulating the immune and anti-inflammatory proteins, resulting in attack by T cells on autoantigens (Morohoshi et al., 2009; Keino et al., 2018). This process leads to stimulation of inflammatory mediators and pathways resulting in the production of cytokines and chemokines, such as IL-4, 5, 6, 8, 10, 13, 17, TGF-beta, IFN-Y, MCP-1, and VEGF. Various inflammatory cytokines were reportedly raised in the serum, fluids, systematically, or in the ocular tissues of AMD patients. Investigations of blood samples of the AMD patients showed elevated levels of monocyte chemotactic protein-1 (MCP-1), IL-6 and IL-8 and that monocytes may lead to the progression of AMD (Chakravarthy and Xu, 2016; Grunin et al., 2016). Considering the previous findings and their role in AMD inflammation, IL-6, IL-8, and MCP-1 were prioritized for the current study. Suppression or inhibition of these cytokines and mediators is the key indicator for study of the anti-inflammatory properties of the chosen drug or treatment (Knickelbein et al., 2015).

**FIGURE 3:**
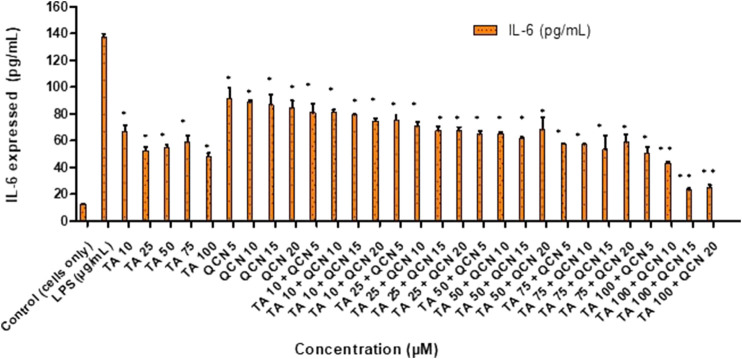
Investigating the anti-inflammatory effect of TA and QCN individually and in combination using IL-6 ELISA: ARPE-19 cells were stimulated using 10 μg/mL LPS for 24 h followed by 24 h treatments. **p < 0.01 (highly significant) *p < 0.05 (significant), n = 2 ± SD.

To induce inflammation, two stimulants were screened; lipopolysaccharide (LPS) and hydrogen peroxide (H_2_O_2_), which had worked effectively in previous studies. LPS is a molecule abundantly available on cell membranes of gram-negative bacteria that can cause inflammatory events by stimulating the release of several cytokines in a vast number of cell types. LPS binds to the CD14 receptor, which exists as membrane protein on ARPE-19 cells leading to the activation of Toll-like-receptor (TLRs) pathways resulting in secretion of inflammatory cytokines (Elner et al., 2003; Kambhampati et al., 2015). Elevated levels of intracellular ROS were observed in the cells exposed to H_2_O_2_ leading to oxidative stress. This reaction leads to the disruption of cellular balance resulting in chronic inflammation (Hussain et al., 2016; Zhang et al., 2020). To investigate the anti-inflammatory effect of both individual drugs and drugs in combination on ARPE-19 cells, ELISAs were used to measure the inflammatory cytokines. Before exposing the cells to stimulants, a cytotoxicity study was carried out to identify any toxic effects of the stimulants on the cells (SF 3). IL-6 and IL-8 inflammatory cytokines secreted by ARPE-19 were analysed by ELISA to select the stimulant and the concentration to be used for further studies to stimulate inflammation. The IL-6 and IL-8 cytokines consistently increased with LPS stimulation from 10 to 50 μg/mL (SF 4(a) and 5). However, in the case of H_2_O_2_ the secretion of IL-6 at 6 and 24 h was similar to control cells (SF 4(b)). Gunawardena et al., claimed that H_2_O_2_ acts as an anti-inflammatory agent by acting as a messenger that can travel through extracellular space and then diffuse into adjacent cells (Gunawardena et al., 2019). In their study, the authors reported the conversion of H_2_O_2_ into water and oxygen in the extracellular space. Given the results observed in the current study, it is possible that H_2_O_2_ did not enter the next cell as a stimulant. Hence there was no significant increase in the secretion of IL-6 cytokine compared to control unstimulated cells. The 10 μg/mL concentration of LPS consistently increased the secretion of IL-6 and IL-8 over 24 h period hence this condition was used for further studies. Similar results were observed by Paeng et al., who investigated the activity of YCG063 (inhibitor of ROS) on inflammation in ARPE-19 (Paeng Hwa et al., 2015). They used LPS to induce the inflammation where LPS at 10 μg/mL consistently secreted IL-6, IL-8, MCP-1, and ICAM-1 cytokines for 24 h stimulation. After stimulating the cells (except for the control unstimulated cells), cells were exposed to different concentrations of TA and QCN individually and in combination to investigate the anti-inflammatory effect (Figure 3).

When compared to LPS stimulated cells, which expresses the maximum amount of IL-6, all the treatments exhibited a significant anti-inflammatory effect by lowering the secretion of cytokine in the ELISA study (*p* < 0.05). This supports the conclusion that corticosteroids such as TA show an anti-inflammatory effect by multiple signal transduction pathways (Barnes, 2006; Imai et al., 2017). In the current study, TA consistently lowered the secretion of IL-6 and showed maximum inhibition at 100 µM. In accordance with previous studies on ARPE-19, QCN decreased the secretion of IL-6 with the maximum effect observed at 20 µM (Hytti et al., 2015). Cheng et al. also reported that QCN at 20 µM showed anti-inflammatory activity on ARPE-19 by lowering the secretion of inflammatory cytokines (IL-6, IL-8, MCP-1, and ICAM-1) (Cheng et al., 2019). They found the lowering of mRNA expression of these cytokines through reverse transcription-quantitative polymerase chain reaction (RT-qPCR) for quercetin treated cells. From the western blot studies, QCN was found to regulate the mitogen-activated protein kinase (MAPK) and nuclear factor kappa B (NF-kB) inflammatory signaling pathways. QCN has the potential to lower the inflammatory responses in retinal pigment epithelial cells and can be considered as a therapeutic agent for inflammation along with its antioxidant properties. As mentioned previously, there is no treatment for multifactorial AMD and monotherapy is not completely effective in treating the disease. In the current study, the efficacy of a novel combination of corticosteroid and flavonoid (TA + QCN) was examined. In accordance with this, different concentrations of TA + QCN were tested for their anti-inflammatory effect. When compared to individual drugs, TA and QCN together displayed a synergetic decrease of IL-6 secretion at higher concentrations (TA 100 + QCN 15 µM and TA 100 + 20 µM) with *p*-value <0.05.

**FIGURE 4:**
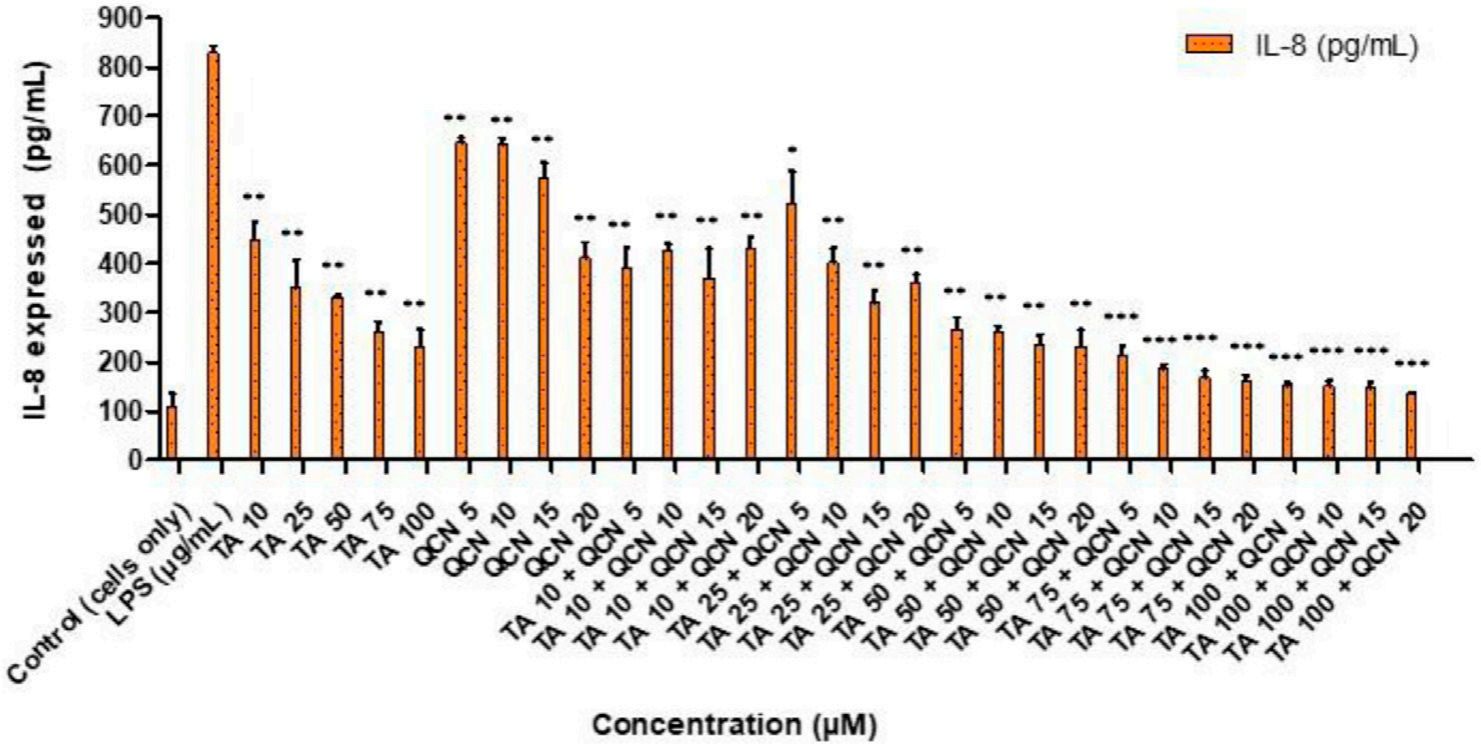
Investigating the anti-inflammatory effect of TA and QCN on their own and in combination using IL-8 ELISA: ARPE-19 cells were stimulated using 10 μg/mL LPS for 24 h followed by 24 h treatments. ***p < 0.001 (very highly significant) **p < 0.01 (highly significant) *p < 0.05 (significant), n = 2 ± SD.

All the treatments significantly reduced IL-8 cytokine secretion when compared to LPS stimulated cells (Figure 4). Whereas the higher concentrations of TA in combination with QCN (TA50 + Q20, TA75 + Q5, 10, 15, 20 and TA100 + Q5, 10, 15, 20) showed the higher anti-inflammatory effect compared to other treatments (*p* < 0.001). Similar to the IL-6 cytokine study, a synergistic effect was observed in some combination concentrations when compared to individual drugs. Specifically, TA at 50 μM and 75 µM in combination with QCN at 10 μM, 15 μM, and 20 μM, as well as TA at 100 µM combined with QCN at 5 μM, significantly decreased IL-8 secretion (*p* < 0.05). The cells treated with individual drugs secreted IL-8 between 232 and 646 pg/mL, whereas the cells treated with combination drugs secreted between 135 and 521 pg/mL (quantified using ELISA).

**FIGURE 5:**
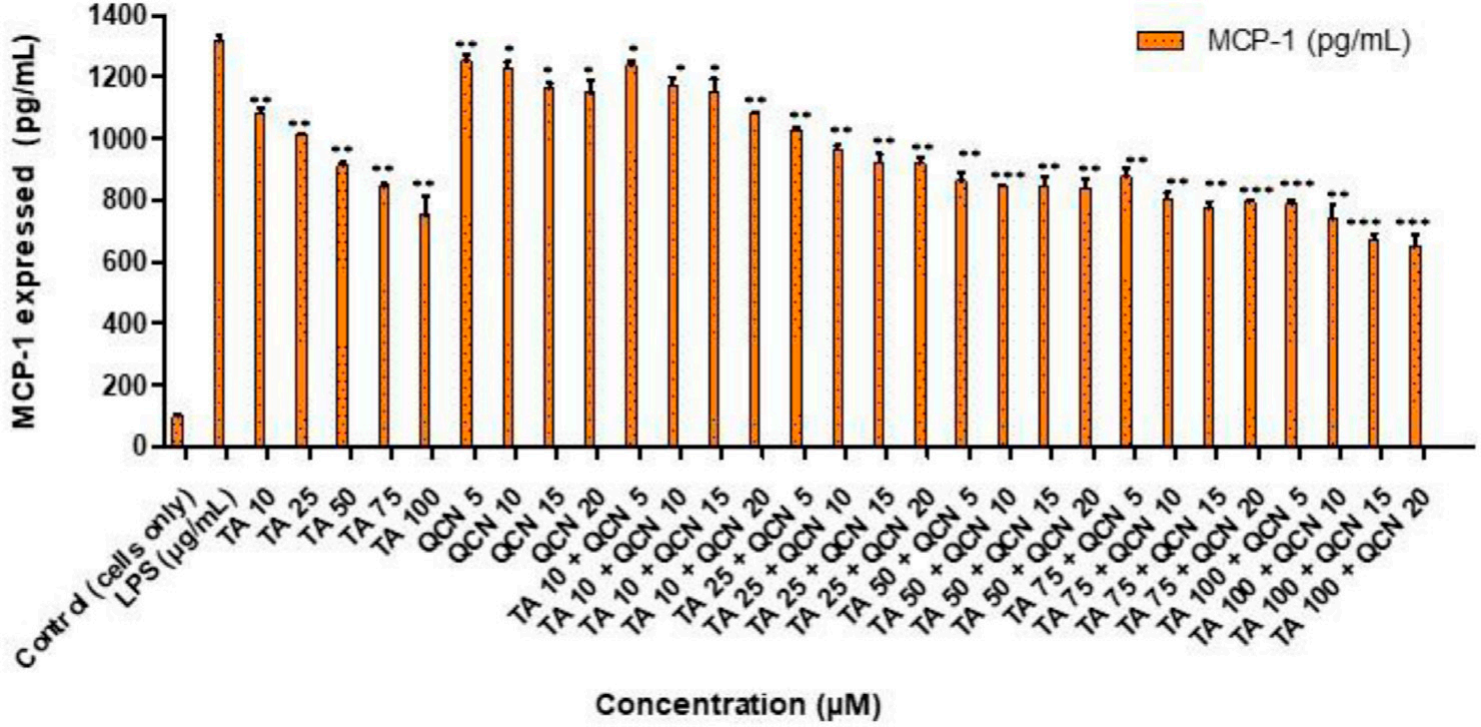
Investigating the anti-inflammatory effect of TA and QCN on their own and in combination using MCP-1 ELISA: ARPE-19 cells were stimulated using 10 μg/mL LPS for 24 h followed by 24 h treatments. ***p < 0.001 (very highly significant) **p < 0.01 (highly significant) *p < 0.05 (significant), n = 2 ± SD.

Following on from the IL-6 and IL-8 cytokines study, MCP-1 was investigated following a similar procedure as a quantitative signal of inflammation. The secretion of MCP-1 cytokine was lowered for all the treatments except for QCN 5 μM, as seen in Figure 5, indicating anti-inflammatory activity. MCP-1 belongs to the chemokine family and acts as a proinflammatory protein which can activate T lymphocytes and monocytes and is reported to be an important indicator of inflammation (Klueh et al., 2016). Dose-dependent decrease of MCP-1 secretion was observed with increase in TA concentration from 10 to 100 µM. The cells treated with higher concentrations of TA 50, 75, and 100 µM secreted MCP-1 between 751 ± 61 to 913 ± 10 pg/mL and in combination with QCN 15 and 20 µM the secretion was further inhibited with a range of 650 ± 38 and 845 ± 30 pg/mL (quantified using MCP-1 ELISA).

As mentioned previously, multiple studies demonstrated that QCN acts as an anti-inflammatory agent by regulating the MAPK pathway in different kinds of cells under various stimulants, whereas TA acts on key inflammatory transcription factors such as NF-κB (Nuclear factor kappa-light-chain-enhancer of activated B cells) (Meng et al., 2018; Min et al., 2007; Bian et al., 2018; C; Li, Zhang, and Frei, 2016; Pitarokoili et al., 2019). As the two therapeutics have different major targets to combat inflammation, the combination therapy had effectively decreased the chosen cytokines secretion in the current study.

### 3.3 Anti-VEGF activity

**FIGURE 6:**
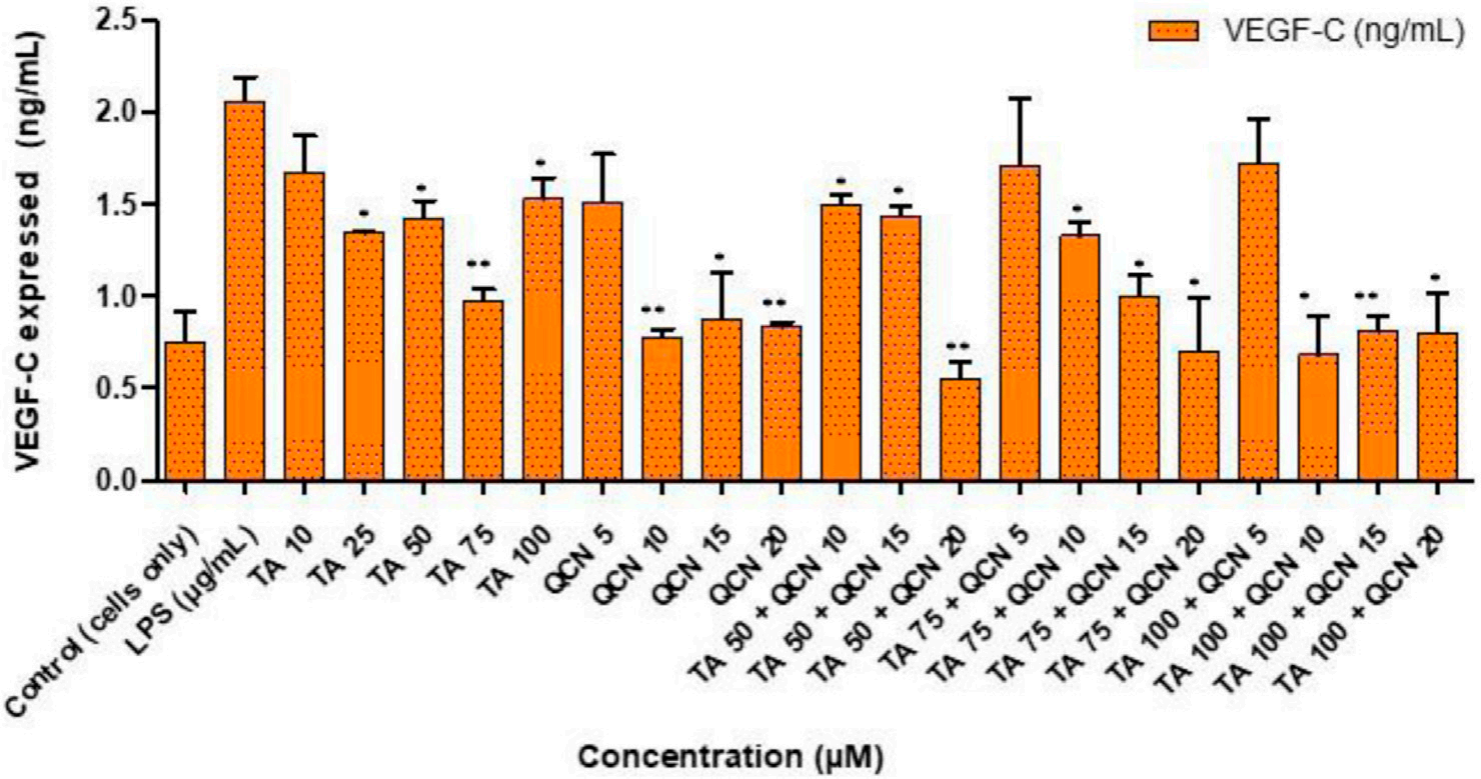
Investigating anti-VEGF activity of TA and QCN on their own and in combination using VEGF-C ELISA: ARPE-19 cells were stimulated using 10 μg/mL LPS for 24 h followed by 24 h treatments. **p < 0.01 (highly significant) *p < 0.05 (significant), n = 2 ± SD.

Neovascularization plays a major role in the progression of AMD hence anti-angiogenic therapies are useful (Pugazhendhi et al., 2021). VEGF is identified to be the major factor in promoting vascular permeability and angiogenesis. This VEGF family includes: VEGF-A, VEGF-B, VEGF-C, VEGF-D, VEGF-E and placenta growth factor (PIGF) (Melincovici et al., 2018). VEGF is secreted in the ocular environment by RPE, endothelial cells and photoreceptors. Elevated levels of VEGF in the vitreous were discovered in AMD patients with neovascularization (Nobl et al., 2016). This increase of VEGF levels will lead to damage of the blood retinal barrier, branching of blood vessels and may also stimulate inflammation by induction of inflammatory mediators like intercellular adhesion molecule-1 (Penn et al., 2008). Due to these factors VEGF is the target for current treatments and considered as a pharmaceutical target for identifying potential treatment for AMD (Kaiser et al., 2021). Considering the findings from anti-inflammatory studies, the drug combination concentrations were filtered down and only higher concentrations of TA (75 and 100 µM) were tested in combination with higher concentrations of QCN (15 and 20 µM) on VEGF-C. Even though all the VEGF proteins in the VEGF family were raised in human plasma during AMD, significantly increased levels of VEGF-C is found in all the layers of the retina, and it correlates with other VEGF proteins (in terms of expression and suppression) (Teague et al., 2016; Teague et al., 2017). Considering these findings for the current study, VEGF-C angiogenic cytokine was prioritised to assess the anti-VEGF activity of the combination therapy. The individual drugs, and combinations thereof, lowered the expression of VEGF-C when compared to LPS stimulated cells, as seen in Figure 6. However, for a few concentrations the decrease was not significant (TA 10 μM, QCN 5 μM, TA 75 µM and TA 100 µM in combination with QCN 5 µM).

QCN demonstrated further suppression of VEGF-C, by lowering the concentration from 2 ± 2 pg/mL (control - LPS stimulated) to 1 ± 0.02 pg/mL (QCN 20 µM). In a previous study investigating the anti-cancer properties of QCN, it was shown to inhibit the pathways (protein kinase B (Akt), mammalian target of rapamycin (mTOR), and ribosomal protein S6 kinase (p70S6K)), which activates VEGF receptors (Pratheeshkumar et al., 2012). This capability of QCN to act on the pathways which secrete VEGF proteins could be the reason for the significant suppression of VEGF-C at higher concentrations (*p* < 0.05). In a study on humans, upon oral administration of QCN it suppressed neovascularization, and the authors also reported visual and retinal restoration (similar to the currently available anti-VEGF therapies) (Richer et al., 2013; Richer et al., 2014). The inflammatory pathways on which QCN acts was studied previously but the exact mechanism of action of anti-VEGF activity is still under investigation (the mechanism behind inhibiting VEGF signaling pathways). Similar to QCN, TA’s anti-VEGF mechanism still needs elucidation but some studies report that it might decrease synthesis of VEGF by non-genomic destabilization of VEGF mRNA (H. Zhou et al., 2012; Sears and George, 2005). As observed in the anti-inflammatory study, combination treatment containing higher concentrations of TA and QCN were effective.

### 3.4 Antioxidant activity

Reactive oxygen species (ROS) levels are monitored to maintain homeostasis at a cellular level. Oxidative stress is a condition where these ROS levels elevate and gather to an extent that leads to the damage of cellular macromolecules and induced apoptosis (Birben et al., 2012). The risk factors which lead to the progression of AMD include: genetics, aging, ethnicity and environmental factors such as high fat diet, smoking and light induced oxidative stress (Datta et al., 2017). According to Harman’s free radical theory of aging, the build-up of free radicals over a life time leads to oxidative damage of cellular macromolecules effecting the physiological condition of the organism (Harman, 1955). Exposure to blue light and ultraviolet rays leads to the degeneration of mitochondria of RPE cells resulting in reduction of ATP generation and increase of ROS levels (Noell et al., 1966; Ivanov et al., 2018). The redox homeostasis of RPE depends on the stimulation of the transcription factor nuclear factor erythroid 2–related factor 2 (Nrf2) and NF-kB and this natural process is effected by the above mentioned factors leading to the oxidative stress observed in AMD (Bellezza et al., 2018). Considering the role of oxidative stress in AMD, the potential therapeutics under investigation in the current study were tested to assess their antioxidant activity using DPPH and DCFH-DA assays.

#### 3.4.1 DPPH assay

**FIGURE 7:**
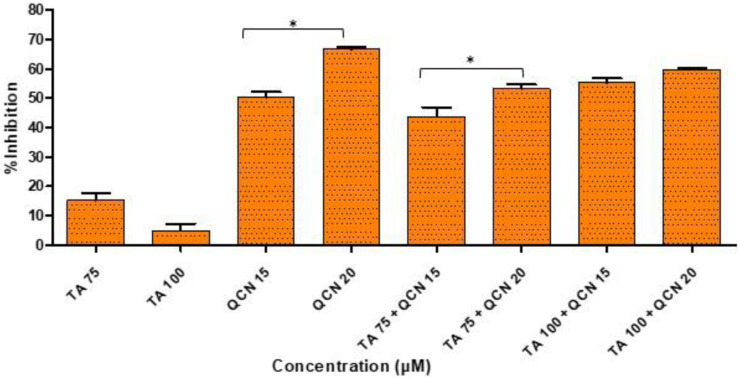
Investigation of antioxidant activity using a DPPH assay by considering the %inhibition of DPPH free radical agent. *p < 0.05 (significant), n = 3 ± SD.

The DPPH assay is one of the most commonly used colorimetric assays and gives an indication of the radical scavenging ability of the test compound. DPPH is a very stable free radical and when it comes in contact with an antioxidant it loses its free radical property resulting in a colour change from violet to yellow (Gonçalves et al., 2018). The higher concentrations of TA and QCN, and in combination, consistently lowered inflammatory cytokine expression and VEGF-C, hence these concentrations were selected to investigate their antioxidant activity (Figure 7).

As expected, QCN efficiently decreased the level of DPPH free radical and exhibited a strong anti-oxidant effect as observed in previous studies, whereas TA displayed a minimal anti-oxidant effect (Hatahet et al., 2017; Caddeo et al., 2019). A higher concentration of QCN (20 µM) demonstrated an enhanced anti-oxidant effect compared to 15 µM by significantly inhibiting DPPH by 67% (*p* < 0.05). QCN 20 µM in combination with TA 75 and 100 µM displayed enhanced radical scavenging activity than QCN 15 µM in combination with TA 75 and 100 µM. The anti-inflammatory effect of flavonoids like QCN primarily depends on their potential to scavenge ROS. This results in regulating of the Nrf2 and NF-kB pathways to maintain cellular homeostasis and prevent oxidative stress (Murakami et al., 2015). QCN on its own and in combination demonstrated increased antioxidant activity when compared to TA. It can regulate both enzyme-mediated and non-enzyme-dependent antioxidant defence systems. It can also activate antioxidant defence systems and maintain oxidative balance by regulating signal pathways such as NRFB (nuclear factor E2-related factor), AMPK (AMP-activated protein kinase), and MAPK (Mitogen-activated protein kinase) (Xu et al., 2019).

#### 3.4.2 DCFH-DA assay

**FIGURE 8:**
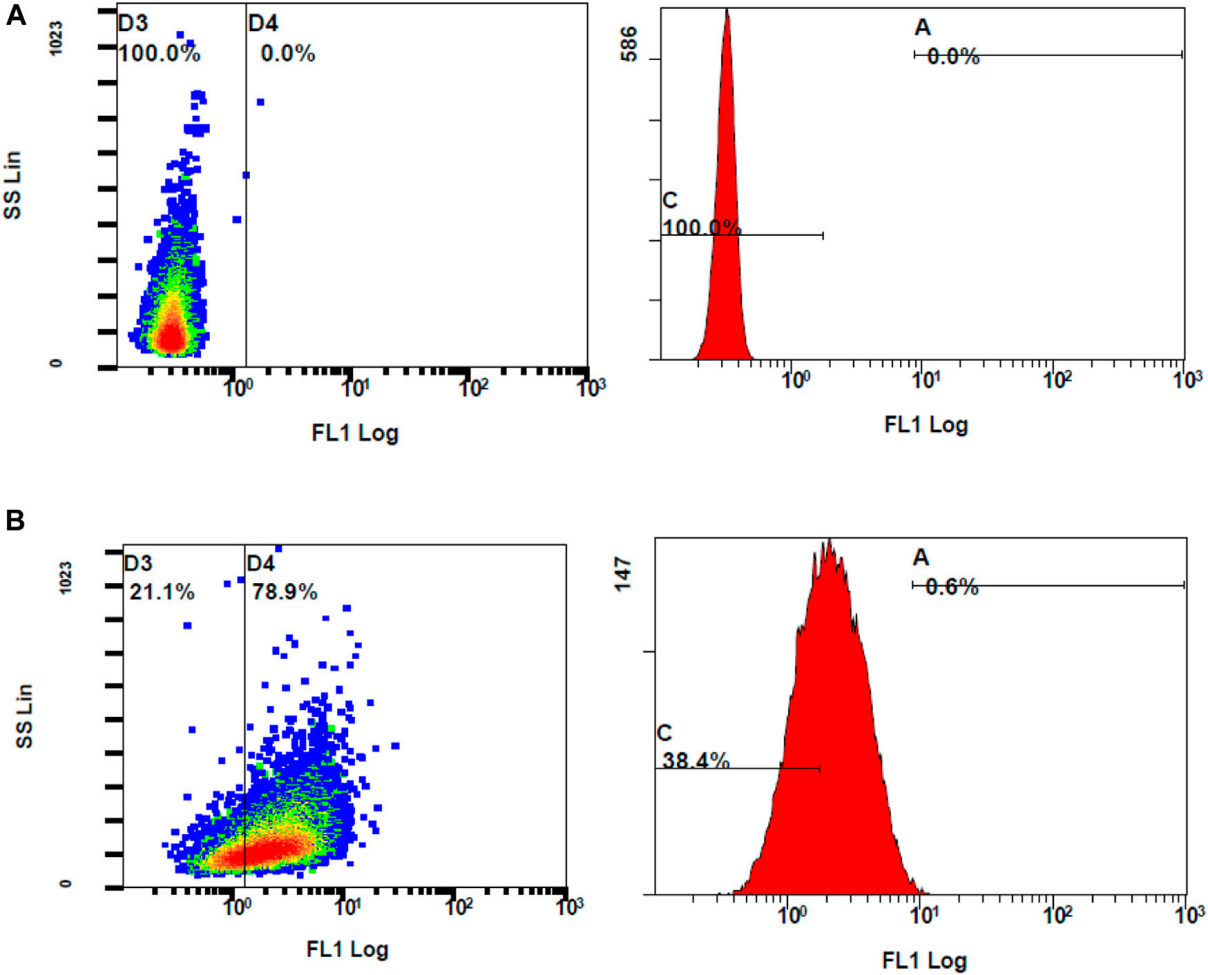
Flow cytometer analysis of ROS generation using DCFH-DA dye **(A)** unstimulated stained cells **(B)** Stimulated stained cells with 300 M hydrogen peroxide.

Following the chemical antioxidant assay (DPPH), the intracellular antioxidant assay was studied to investigate the effectiveness of the treatments on the stressed ARPE-19 cells. For the 2′,7′-dichlorodihydrofluorescein diacetate (DCFH-DA) assay, oxidative stress was induced to ARPE-19 cells using stimulants. Upon stimulation and treatment, the intracellular ROS levels were measured using flow cytometry to assess the anti-oxidant effect of the treatments. The oxidation of DCFH-DA to 2′-7′dichlorofluorescein (DCF) was used for the detection of intracellular ROS levels including nitrogen dioxide and hydroxyl radicals. The cells will take up the DCFH-DA dye where cellular esterase cleaves off the acetyl groups, resulting in the formation of DCFH (2′,7′-dichlorodihydrofluorescein). Oxidation of DCFH in the presence of intracellular ROS leads to the formation of DCF. This DCF fluorescent molecule can be detected using a flow cytometer by mean fluorescence intensity (MFI). To induce oxidative stress, LPS and H_2_O_2_ were tested. When compared to unstimulated cells, the cells stimulated with H_2_O_2_ at 300 µM induced oxidative stress, which is reflected by stained cells and MFI as seen in Figure 8.

**FIGURE 9:**
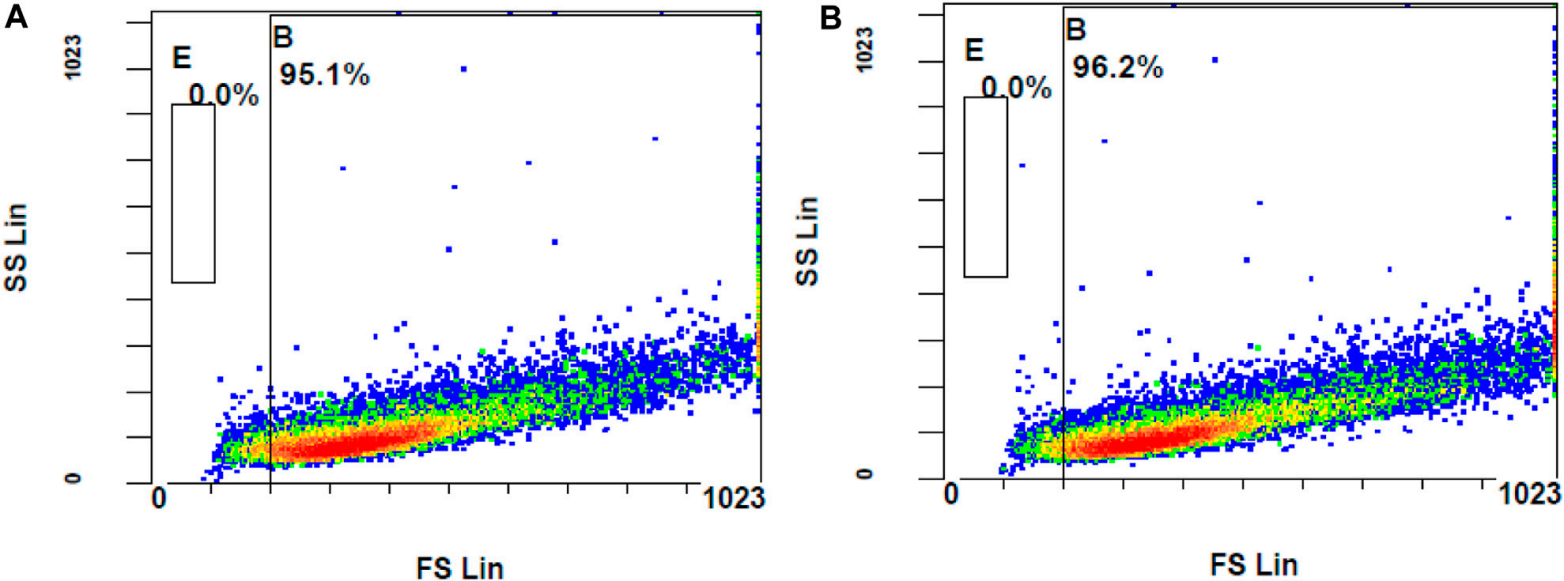
Comparison of unstimulated **(A)** stained and **(B)** unstained ARPE-19 cells using flow cytometer analysis.

The ROS levels of the cells stimulated with H_2_O_2_ at 300 µM had significantly increased when compared to unstimulated cells (MFI of unstimulated stained and stimulated stained cells was 2.01 ± 0.10 and 3.73 ± 0.29, respectively, with a *p*-value <0.05). Considering the MFI value and the appearance of stained cells in the fluorescent gate, 300 µM concentration of H_2_O_2_ was selected for further investigation. To determine the effect of DCFH-DA dye on the morphology or the characteristics of the cells, flow cytometry analysis was performed on the control unstimulated unstained and control unstimulated stained cells (Figure 9). There were no changes of the ARPE-19 cells were observed with the exposure to the DCFH-DA dye.

**FIGURE 10:**
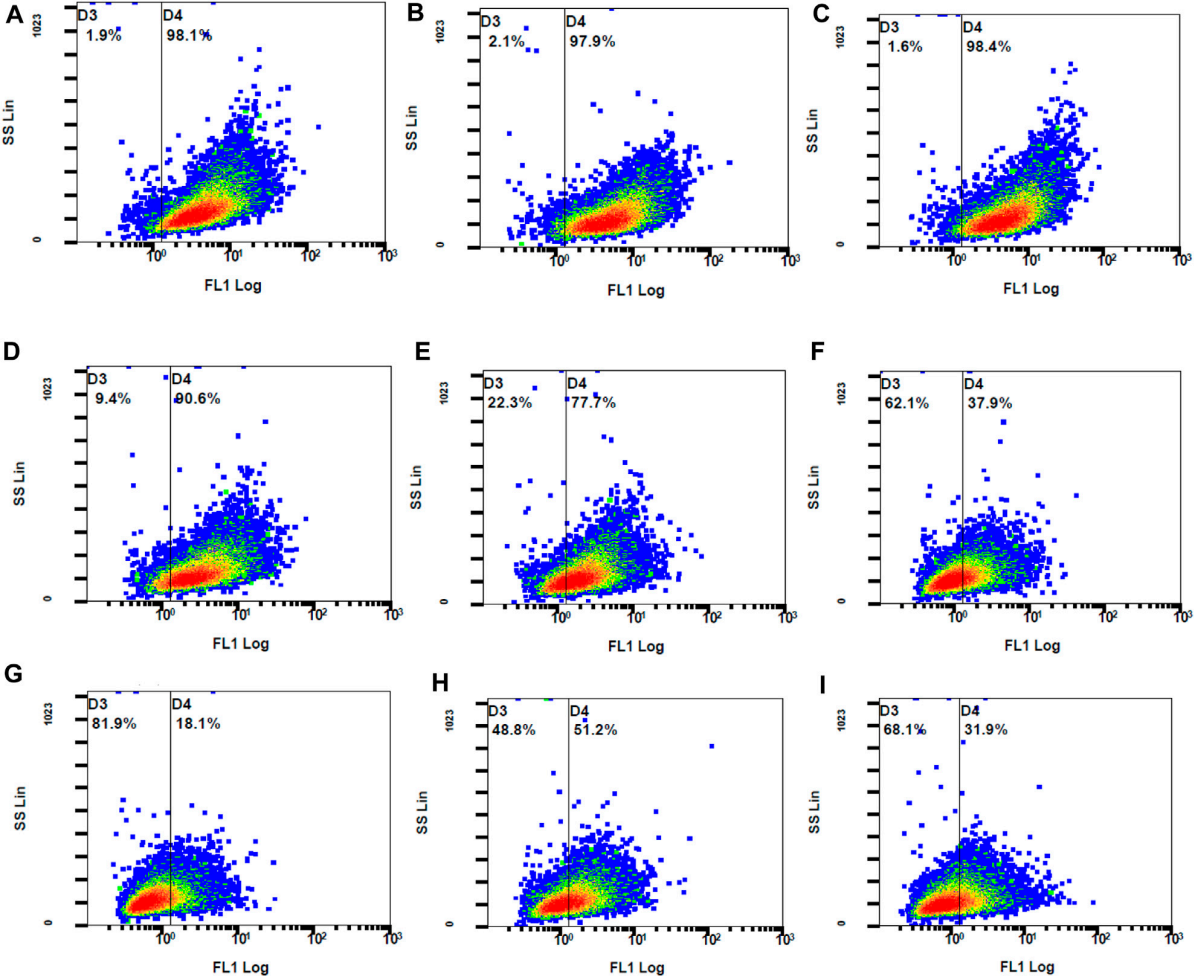
The fluorescent ARPE-19 cells were in gate D4 and the non-fluorescent cells in gate D3 **(A)** control stimulated **(B)** TA 75 µM **(C)** TA 100 µM **(D)** Q 15 µM **(E)** Q 20 µM **(F)** TA 75 + Q 15 µM **(G)** TA 75 + Q 20 µM **(H)** TA 100 + Q 15 μM, and **(I)** TA 100 + Q 20 µM.

The concentrations used for the DPPH assay were those used for the investigation of the intracellular ROS levels. When compared to the control stimulated stained, QCN and all the combination concentrations significantly suppressed the generated ROS. The findings of the DCFH-DA were similar to the DPPH anti-oxidant assay where TA did not lower the ROS levels. The combination drugs exhibited a synergetic anti-oxidant effect by significantly reducing the ROS, which was represented by lower MFI values (*p*-value <0.05) when compared to individual drugs (SF six and 7). The graphs in Figure 10 represent the stained cells with ROS in Gate D4 (fluorescent gate) and unstained cells in gate D3 (non-fluorescent gate).

**FIGURE 11:**
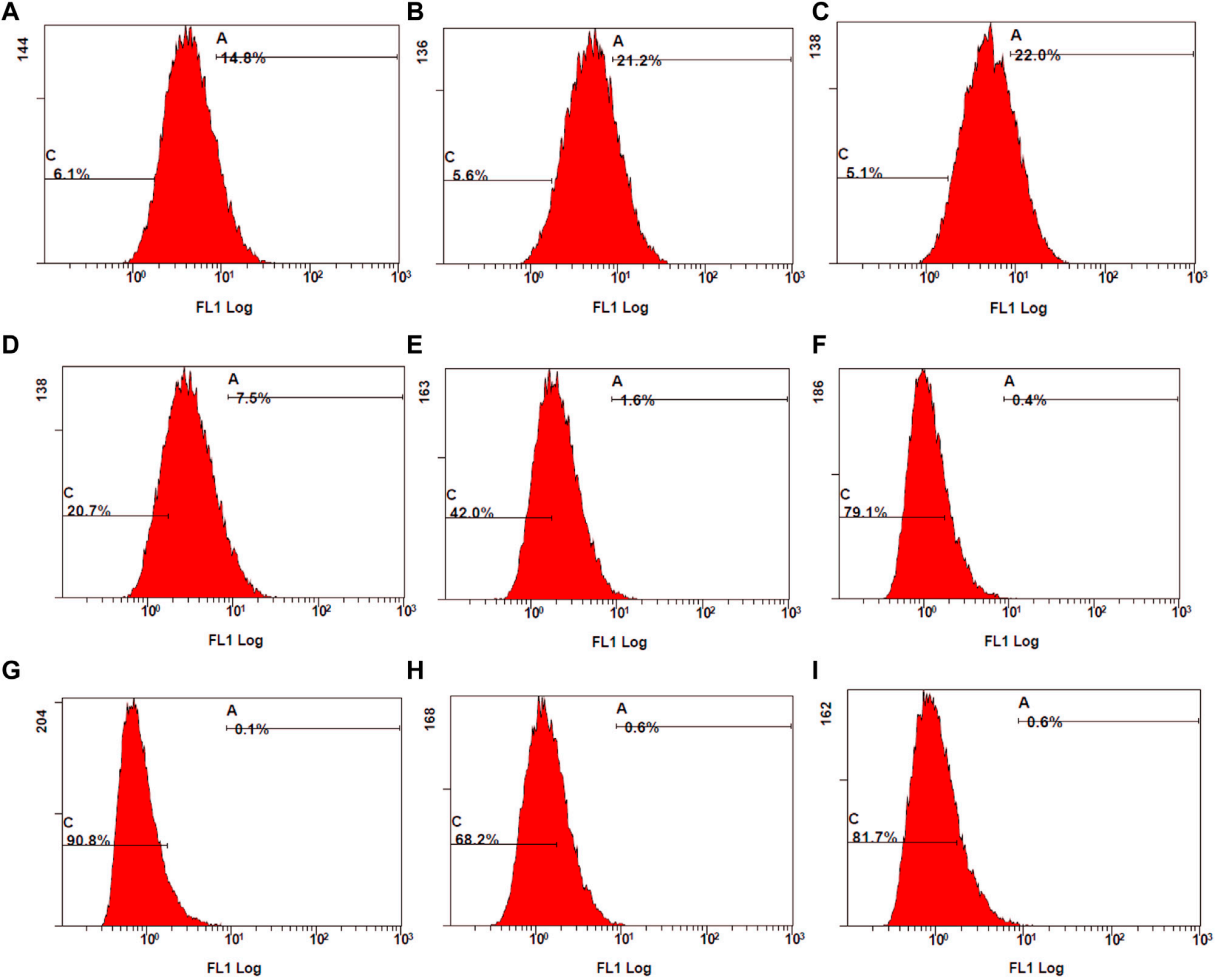
Examination of stained ROS species by mean fluorescence intensity (MFI) measured using flow cytometry. **(A)** control stimulated **(B)** TA 75 µM **(C)** TA 100 µM **(D)** Q 15 µM **(E)** Q 20 µM **(F)** TA 75 + Q 15 µM **(G)** TA 75 + Q 20 µM **(H)** TA 100 + Q 15 μM, and **(I)** TA 100 + Q 20 µM.

The control stimulated stained cells displayed 98.1% cell population in the fluorescent gate indicating the generation of ROS due to oxidative stress. Due to the synergetic antioxidant effect the stained cells containing ROS was significantly reduced, which is represented by a shift of cell population to the non-fluorescent gate. The cells containing ROS were reduced to 18%–51%, which was also represented by the increase in non-stained cells between 49% and 82%. In accordance with the stained and unstained cell population in gates D4 and D3, the fluorescent peak shifts from 10^1^ to 10^0^ highlight the strong anti-oxidant effect by reducing the intracellular ROS (Figure 11).

Suntiparpluacha et al. studied the effect of TA and TA in combination with vitamin C supplementation on oxidative stress in human chondrocytes (Suntiparpluacha et al., 2016). In their study, TA did not show any anti-oxidant effect and exhibited a slight increase in oxidative stress. When they combined TA with an antioxidant (vitamin C), the oxidative stress was further decreased compared to the antioxidant alone. The outcome of the current study was similar to their findings, the addition of QCN could decrease the side effects of TA and enhance the anti-oxidant activity of QCN. In previous studies corticosteroids such as dexamethasone and cortisone have shown a decrease in antioxidant enzymes and mitochondrial activity in hippocampal neurons and rat pheochromocytoma cell lines (You et al., 2009; Tang et al., 2013). These side effects of corticosteroids might be reduced with the addition of anti-oxidant agents such as QCN.

The retina has a high metabolism, and the oxidative stress effects are high as the ROS are generated by the mitochondria of the cells. The generation of ROS hinders the physiological process of the cells leading to inflammation, autophagy, and apoptosis (Tisi et al., 2021). Monitoring the ROS levels is the key factor for retinal diseases, such as AMD, diabetic retinopathy, and retinitis pigmentosa, etc. In both the antioxidant assays, QCN alone and in combination with TA showed effectiveness in reducing the ROS levels and proved to be an effective antioxidant agent.

## 4 Conclusion

The critical factor in AMD is its multifactorial nature, activation of inflammatory and VEGF signalling pathways leading to the secretion of cytokines with parallel oxidative stress affecting the physiological process by the generation of ROS. Treating these multiple conditions to protect the retina and restore the vision of people suffering from AMD is a great challenge for the scientific community. As mentioned previously although the current treatments are efficient in delaying the progression of the disease, they do not provide a cure. Hence investigating new therapeutics and delivering them through non-invasive routes is the aim of the current study.

Firstly, the individual drug concentrations (TA and QCN) and the combinations (TA + QCN) were safe on the retinal cell line and displayed no signs of synergetic toxicity or changes in morphology of the cells at the chosen concentrations. The combination exhibited an increased anti-inflammatory effect as the TA and QCN act predominantly on different inflammatory signalling pathways (TA acts on NF kappa B and QCN on MAPK). In terms of anti-VEGF activity, both drugs act in a different way, QCN inhibits the kinase pathways leading to deactivation of VEGF receptors whereas TA destabilises VEGF mRNA, which lead to the suppression of VEGF-C with the combination treatments. Both the anti-oxidant assays (DPPH and DCFH-DA) showed similar outcomes by exhibiting the synergetic effect in reducing ROS levels when treated with combination drugs. All these findings suggest corticosteroid (TA) and flavonoid (QCN) as a potential combination therapy to target AMD.

## Data Availability

The original contributions presented in the study are included in the article/Supplementary Material, further inquiries can be directed to the corresponding author.
