# Supplementary material for: Investigation of novel combination therapy for age-related macular degeneration on ARPE-19 cells
Source: Front Drug Deliv. 2024 Apr 19;4:1337686. doi: 10.3389/fddev.2024.1337686 (PMC12363285; doi:10.3389/fddev.2024.1337686)
Supplement: Supplementary file 1 [file DataSheet1.docx]

Investigation of a novel combination therapy for age-related macular degeneration on ARPE-19 cells

# Madhuri Dandamudi ^1^*, Peter McLoughlin ^1^, Gautam Behl ^2^, Lee Coffey ^1^, Anuj Chauhan ^3^, David Kent ^4^, (Sweta Rani ^1^ and Laurence Fitzhenry ^1^)* †

1. Ocular Therapeutics Research Group, Pharmaceutical and Molecular Biotechnology Research Centre, Department of science, South East Technological University, X91 K0EK Waterford, Ireland.
2. Eirgen Pharma Ltd, Ireland

^3^ Department of Chemical and Biological Engineering, Colorado School of Mines, Colorado, CO 80401, USA; [chauhan@mines.edu](mailto:chauhan@mines.edu)

^4^ The Vision Clinic, R95 XC98 Kilkenny, Ireland; [dkent@liverpool.ac.uk](mailto:dkent@liverpool.ac.uk)

†These authors contributed equally to this work and share last authorship

*** Correspondence:**Corresponding Author: Madhuri Dandamudi
Madhuri.dandamudi@setu.ie

## Supplementary Figures

(a)

(b)

Supplementary Figure 1: % Cell viability of (a) TA and (b) QCN for a range of concentrations between 10 and 250 µM. Data points represent the average ± SD of n = 3 biological replicates for (a) and technical replicates for (b).

Supplementary Figure 2: % Cell viability of QCN for a range of concentrations between 1 and 100 µM, data points represent the average ± SD of n = 3 ± SD biological replicates.

(a)

(b)

Supplementary Figure 3: Assessment of cytotoxicity of (a) LPS and (b) hydrogen peroxide on ARPE-19 cells up to 48 h, n=3 ± SD.

(a) (b)

Supplementary Figure 4: Secretion of IL-6 by ARPE-19 on stimulation with different concentrations of (a) LPS and (b) hydrogen peroxide, n=3 ± SD.

Supplementary Figure 5: Levels of IL-8 expressed by ARPE-19 upon stimulation with LPS, n=2 ± SD.

Supplementary Figure 6: Investigation of intracellular ROS levels by estimating mean fluorescence intensity (MFI) using flow cytometer. ** P <0.01 (highly significant) in comparison with control stimulated stained (Control SS) cells. n=3 ± SD.

Supplementary Figure 7: Investigation of intracellular ROS levels by estimating mean fluorescence intensity (MFI) using a flow cytometer. Reduction in MFI with combination drugs treatment is highlighted in deep orange.
